# Palladium-Catalyzed C–H Arylation of 1,2,3-Triazoles

**DOI:** 10.3390/molecules21101268

**Published:** 2016-09-22

**Authors:** Chengwei Zhang, Lin You, Chuo Chen

**Affiliations:** Department of Biochemistry, UT Southwestern Medical Center, Dallas, TX 75390, USA; ZCW574562@gmail.com (C.Z.); Lin.You@UTSouthwestern.edu (L.Y.)

**Keywords:** C–H arylation, palladium, triazole

## Abstract

Palladium(II) acetate, in combination with triphenylphosphine, catalyzes direct arylation of 1,4-disubstituted 1,2,3-triazoles effectively. This C–H arylation reaction provides facile access to fully substituted triazoles with well-defined regiochemistry.

## 1. Introduction

1,2,3-Triazole has attracted increasing attention in medicinal chemistry and material sciences because of the recent development of transition metal-catalyzed Huisgen 1,3-dipolar cycloaddition of azides and terminal alkynes [1,2,3,4,5,6,7]. In contrast to the thermal process that is not regioselective, the copper(I)- and ruthenium(II)-catalyzed methods afford 1,4- and 1,5-disubstituted 1,2,3-triazoles, respectively [8,9,10,11,12,13]. Methods such as in situ cross-coupling or transmetallation of the triazolyl cuprate intermediate have also been developed to allow for the synthesis of 1,4,5-trisubstituted 1,2,3-triazoles with well-defined regiochemistry [14,15,16,17,18,19,20,21]. However, C–H functionalization of disubstituted triazoles is arguably the most versatile and convenient way to construct 1,4,5-trisubstituted 1,2,3-triazoles [22,23,24,25,26,27]. During a recent structure–activity relationship study of a triazole-class of small-molecule Wnt inhibitors, we found that the reported C–H arylation methods gave very low yields of the coupling products. We report herein the identification of new coupling conditions that supported the synthesis of fully substituted 1,2,3-triazoles.

## 2. Results

Our study commenced with the optimization of the reaction parameters for coupling triazole **1** with phenyl bromide, as the reported methods gave a yield of only <20% of the arylation product **2** (Table 1, entries 1 and 2) [22,23]. Based on our experience in heterocycle C–H arylation [28], we chose the concerted metalation-deprotonation (CMD)-type palladium-catalyzed method [29,30,31,32,33,34,35,36,37]. After a brief survey of reaction parameters based on Gevorgyan’s protocol [22], we focused on studying the effects of ligand and base (Table 1). We found that potassium carbonate was a more effective base (entries 3–5), and triphenylphosphine promoted the coupling reaction to give **2** with significantly increased yields (entries 5–13). Switching the solvent to toluene further improved the conversion (entry 14).

With suitable coupling conditions in hand, we tested the scope of this C–H arylation reaction (Table 2). The coupling of **3** with aryl bromide proceeded well except for a few cases. In general, 4-substituted aryl bromides reacted smoothly regardless of their electronic properties (entries 1–5). Only 4-fluorophenyl bromide reacted with **1** slowly (entry 6). However, this coupling reaction is sensitive to electronic perturbation at the 3-position of the aryl bromide. Although introduction of a slightly electron-rich methyl group did not affect the coupling efficiency (entry 7), the presence of an electron-withdrawing aldehyde group resulted in the formation of multiple by-products (entry 8). The reaction is also sensitive to steric perturbation at the 2-position of the aryl bromide. Coupling of **1** with 2-methoxyphenyl bromide gave a good yield of the desired product (entry 9), but the reaction of **1** and 2-bromotoluene proceeded with a modest conversion (entry 10). The coupling of **1** and 1-naphthyl bromide was also slightly slower (entry 11).

C–H arylation of various other triazoles also proceeded well. There is no reduction in coupling efficiency for 3-pyridyl, 2-pyridyl, and phenyl-substituted triazoles **3** (entries 12–14). However, the introduction of a 4-methoxyl or a 2-trifluoromethyl group at the C4-position led to decreased yields of **4** (entries 15 and 16). The reaction of 2-trifluoromethylphenyl substituted triazole **3** with phenyl bromide also gave a 29% yield of the corresponding α-arylation product [38,39,40], in addition to the desired C–H arylation product. No reaction occurred with α-substituted triazoles **3** due to the congested environment around the triazole C–H. Performing the reaction with microwave-heating at a slightly higher temperature gave a small amount of products, but a prolonged reaction time led to significant decomposition (entries 17 and 18).

## 3. Materials and Methods

### 3.1. General Methods

All reactions were performed in glassware under a positive pressure of argon. Flash column chromatography was performed on a Teledyne ISCO CombiFlash Rf 200 system (Isco, Inc., Lincoln, NE, USA) using silica gel 60 (230–400 mesh). Thin layer chromatography (TLC) analyses were performed on EMD 250 μm Silica Gel 60 F254 plates (Merck KGaA, Darmstadt, Germany) and visualized by quenching of UV fluorescence (λ_max_ = 254 nm) or by staining with ceric ammonium molybdate. ^1^H-NMR spectra were recorded on a Varian Inova-400 instrument (Varian, Inc., Palo Alto, CA, USA). Chemical shifts are reported in ppm (δ) relative to the residual solvent signals of the solvent (δ 7.26 for CHCl_3_), coupling constants (*J*) are reported in Hz and the multiplicities are presented as follows: s = singlet, brs = broad singlet, d = doublet, t = triplet, q = quartet, and m = multiplet. Mass spectra were acquired on Agilent 6120 Single Quadrupole Liquid Chromatography tandem Mass Spectrometer (LC/MS) (Agilent Technologies, Santa Clara, CA, USA). High-resolution mass spectrum was acquired by the Mass Spectrometry Facility at the University of Austin.

### 3.2. General Precedure for C–H Arylation and Compound Characterization

Palladium(II) acetate (10 mol %), triphenylphosphine (20 mol %), potassium carbonate (2.0 equiv.) and aryl bromide (3.0 equiv.) were added to a solution of triazole **3** (1.0 equiv.) in toluene. After stirring for 24 h at 120 °C, the mixture was quenched with saturated ammonium chloride and extracted with ethyl acetate. The combined organic layers were washed with brine and dried over sodium sulfate. The solvent was removed under a vacuum, and the residue was purified by flash column chromatography on silica gel to provide **4**.

*tert-Butyl 2-(5-phenyl-4-(pyridin-4-yl)-1H-1,2,3-triazol-1-yl)acetate (***2***).* White solid; IR (neat, cm^–1^) 3402, 2219, 1615, 1506, 1456, 1368, 1236, 1157, 1048; ^1^H-NMR (400 MHz, CDCl_3_) δ 8.52 (brs, 2H), 7.59–7.55 (m, 5H), 7.37–7.34 (m, 2H), 4.90 (s, 2H), 1.39 (s, 9H); ^13^C-NMR (100 MHz, CDCl_3_) δ 165.3, 150.0, 141.6, 138.3, 136.2, 130.4, 129.6, 129.5, 126.6, 120.5, 83.6, 49.9, 27.8; High resolution mass spectrometry (HRMS)-electrospray ionization (ESI) (*m*/*z*): calcd. for C_19_H_21_N_4_O_2_ [M + H]^+^ 337.1659, found 337.1666.

*tert-Butyl 2-(5-(4-methoxyphenyl)-4-(pyridin-4-yl)-1H-1,2,3-triazol-1-yl)acetate.* White solid; ^1^H-NMR (400 MHz, CDCl_3_) δ 8.49 (brs, 2H), 7.50 (d, *J* = 5.2 Hz, 2H), 7.25 (d, *J* = 8.4 Hz, 2H), 7.03 (d, *J* = 8.4 Hz, 2H), 4.87 (s, 2H), 3.88 (s, 3H), 1.40 (s, 9H); ^13^C-NMR (100 MHz, CDCl_3_) δ 165.5, 161.3, 149.6, 141.6, 139.2, 136.4, 131.2, 120.7, 118.2, 115.2, 83.8, 55.6, 49.9, 28.0; MS-ESI (*m*/*z*): calcd. for C_20_H_23_N_4_O_3_ [M + H]^+^ 367.2, found 367.2.

*tert-Butyl 2-(5-(4-ethoxycarbonylphenyl)-4-(pyridin-4-yl)-1H-1,2,3-triazol-1-yl)acetate*. White solid; ^1^H-NMR (400 MHz, CDCl_3_) δ 8.51 (brs, 2H), 8.20 (d, *J* = 8.0 Hz, 2H), 7.49 (d, *J* = 4.8 Hz, 2H), 7.45 (d, *J* = 8.4 Hz, 2H), 4.90 (s, 2H), 4.44 (q, *J* = 7.2 Hz, 2H), 1.44–1.39 (m, 12H); ^13^C-NMR (100 MHz, CDCl_3_) δ 165.6, 165.2, 149.2, 141.8, 139.2, 135.7, 132.7, 131.1, 130.8, 130.0, 121.0, 84.2, 61.8, 50.1, 28.0, 14.4; MS-ESI (*m*/*z*): calcd. for C_22_H_25_N_4_O_4_ [M + H]^+^ 409.2, found 409.2.

*tert-Butyl 2-(4-(pyridin-4-yl)-5-(4-(trifluoromethyl)phenyl)-1H-1,2,3-triazol-1-yl)acetate.* White solid; ^1^H-NMR (400 MHz, CDCl_3_) δ 8.52 (brs, 2H), 7.79 (d, *J* = 8.0 Hz, 2H), 7.51 (d, *J* = 8.0 Hz, 2H), 7.41(d, *J* = 4.8 Hz, 2H), 4.90 (s, 2H), 1.38 (s, 9H); ^13^C-NMR (100 MHz, CDCl_3_) δ 165.2, 150.2, 142.2, 138.1, 134.8, 132.9, 132.5, 130.7, 130.5, 126.7(q, *J* = 3.7 Hz), 125.0, 122.2, 120.9, 84.2, 50.2, 27.9; MS-ESI (*m*/*z*): calcd. for C_20_H_20_F_3_N_4_O_2_ [M + H]^+^ 405.2, found 405.2.

*tert-Butyl 2-(5-(4-cyanophenyl)-4-(pyridin-4-yl)-1H-1,2,3-triazol-1-yl)acetate.* White solid; ^1^H-NMR (400 MHz, CDCl_3_) δ 8.55 (brs, 2H), 7.84 (d, *J* = 8.0 Hz, 2H), 7.53 (d, *J* = 8.4 Hz, 2H), 7.45 (d, *J* = 4.4 Hz, 2H), 4.91 (s, 2H), 1.40 (s, 9H); ^13^C-NMR (100 MHz, CDCl_3_) δ 165.1, 149.4, 142.2, 138.8, 134.7, 133.4, 131.6, 130.8, 121.1, 117.7, 114.9, 84.5, 50.2, 28.0; MS-ESI (*m*/*z*): calcd for C_20_H_20_N_5_O_2_ [M + H]^+^ 362.2, found 362.2.

*tert-Butyl 2-(5-(4-fluorophenyl)-4-(pyridin-4-yl)-1H-1,2,3-triazol-1-yl)acetate.* White solid; ^1^H-NMR (400 MHz, CDCl_3_) δ 8.51 (brs, 2H), 7.49 (d, *J* = 4.8 Hz, 2H), 7.37–7.34 (m, 2H), 7.26–7.22 (m, 2H), 4.88 (s, 2H), 1.40 (s, 9H); ^13^C-NMR (100 MHz, CDCl_3_) δ 165.3, 148.9, 141.7, 135.8, 132.0 (d, *J* = 8.5 Hz), 130.9, 129.9 (d, *J* = 13.4 Hz), 122.5 (d, *J* = 3.5 Hz), 117.3 (d, *J* = 21.9 Hz), 110.1, 84.1, 50.0, 28.0; MS-ESI (*m*/*z*): calcd. for C_19_H_20_FN_4_O_2_ [M + H]^+^ 355.2, found 355.2.

*tert-Butyl 2-(5-(3-methylphenyl)-4-(pyridin-4-yl)-1H-1,2,3-triazol-1-yl)acetate.* White solid; ^1^H-NMR (400 MHz, CDCl_3_) δ 8.51 (brs, 2H), 7.49 (brs, 2H), 7.42–7.34 (m, 2H), 7.14 (s, 1H), 7.12 (d, *J* = 9.2 Hz, 1H), 4.86 (s, 2H), 2.38 (s, 3H), 1.39 (s, 9H); ^13^C-NMR (100 MHz, CDCl_3_) δ 165.5, 149.9, 141.6, 139.7, 138.8, 136.5, 131.4, 130.2, 129.6, 126.8, 126.6, 120.8, 83.8, 50.0, 27.9, 21.5; MS-ESI (*m*/*z*): calcd. for C_20_H_23_N_4_O_2_ [M + H]^+^ 351.2, found 351.2.

*tert-Butyl 2-(5-(3-formylphenyl)-4-(pyridin-4-yl)-1H-1,2,3-triazol-1-yl)acetate.* White solid; ^1^H-NMR (400 MHz, CDCl_3_) δ 10.06 (s, 1H), 8.10 (d, *J* = 8.0 Hz, 1H), 7.92 (s, 1H), 7.75 (t, *J* = 7.6 Hz, 1H), 7.68–7.62 (m, 2H), 7.54–7.52 (m, 2H), 7.47–7.45 (m, 1H), 4.91 (s, 2H), 1.39 (s, 9H); ^13^C-NMR (100 MHz, CDCl_3_) δ 190.9, 165.3, 150.3, 135.8, 132.3, 132.2, 132.1, 132.1, 130.7, 130.6, 128.7, 128.6, 120.8, 84.3, 50.2, 28.0; MS-ESI (*m*/*z*): calcd. for C_20_H_21_N_4_O_3_ [M + H]^+^ 365.2, found 365.2.

*tert-Butyl 2-(5-(2-methoxyphenyl)-4-(pyridin-4-yl)-1H-1,2,3-triazol-1-yl)acetate.* White solid; ^1^H-NMR (400 MHz, CDCl_3_) δ 8.50 (brs, 2H), 7.57–7.54 (m, 3H), 7.22 (dd, *J* = 8.0, 1.6 Hz, 1H), 7.10–7.06 (m, 2H), 4.89 (dd, *J* = 170.4, 14.8 Hz, 2H), 3.74 (s, 3H), 1.34 (s, 9H); ^13^C-NMR (100 MHz, CDCl_3_) δ 165.2, 157.1, 148.2, 141.6, 140.7, 133.9, 132.7, 132.0, 121.6, 120.9, 114.8, 111.9, 83.6, 55.8, 50.3, 27.9; MS-ESI (*m*/*z*): calcd. for C_20_H_23_N_4_O_3_ [M + H]^+^ 367.2, found 367.2.

*tert-Butyl 2-(5-(2-methylphenyl)-4-(pyridin-4-yl)-1H-1,2,3-triazol-1-yl)acetate.* White solid; ^1^H-NMR (400 MHz, CDCl_3_) δ 8.50 (brs, 2H), 7.50–7.47 (m, 3H), 7.39–7.34 (m, 2H), 7.25 (d, *J* = 6.8 Hz, 1H), 4.81(dd, *J* = 103.6, 17.2 Hz, 2H), 2.00 (s, 3H), 1.36 (s, 9H); ^13^C-NMR (100 MHz, CDCl_3_) δ 165.1, 149.4, 141.5, 139.5, 138.1, 135.9, 131.4, 131.0, 130.2, 127.0, 125.9, 120.1, 83.8, 49.8, 27.9, 19.5; MS-ESI (*m*/*z*): calcd. for C_20_H_23_N_4_O_2_ [M + H]^+^ 351.2, found 351.2.

*tert-Butyl 2-(5-(naphthalen-1-yl)-4-(pyridin-4-yl)-1H-1,2,3-triazol-1-yl)acetate.* White solid; ^1^H-NMR (400 MHz, CDCl_3_) δ 8.40 (brs, 2H), 8.09 (d, *J* = 8.0 Hz, 1H), 7.98 (d, *J* = 8.4 Hz, 1H), 7.62–7.50 (m, 3H), 7.43–7.40 (m, 3H), 7.33 (d, *J* = 8.4 Hz, 1H), 4.76 (dd, *J* = 159.6, 17.2 Hz, 2H), 1.28 (s, 9H); ^13^C-NMR (100 MHz, CDCl_3_) δ 165.2, 149.3, 142.6, 139.0, 134.8, 133.9, 131.4, 129.3, 129.0, 128.1, 127.3, 125.6, 124.4, 123.6, 120.5, 110.1, 83.8, 50.0, 27.8; MS-ESI (*m*/*z*): calcd. for C_23_H_23_N_4_O_2_ [M + H]^+^ 387.2, found 387.2.

*tert-Butyl 2-(5-phenyl-4-(pyridin-3-yl)-1H-1,2,3-triazol-1-yl)acetate.* White solid; ^1^H-NMR (400 MHz, CDCl_3_) δ 8.70 (d, *J* = 2.2 Hz, 1H), 8.49 (dd, *J* = 4.9, 1.7 Hz, 1H), 8.04 (dt, *J* = 8.0, 1.9 Hz, 1H), 7.57–7.48 (m, 3H), 7.37–7.32 (m, 2H), 7.29 (dd, *J* = 7.9, 5.0 Hz, 1H), 4.91 (s, 2H), 1.40 (s, 9H); ^13^C-NMR (100 MHz, CDCl_3_) δ 165.5, 148.9, 147.8, 141.6, 135.2, 134.0, 130.3, 129.8, 129.6, 127.0, 126.8, 123.5, 83.7, 50.1, 27.9; MS-ESI (*m*/*z*): calcd. for C_19_H_21_N_4_O_2_ [M + H]^+^ 337.2, found 337.2.

*tert-Butyl 2-(5-phenyl-4-(pyridin-2-yl)-1H-1,2,3-triazol-1-yl)acetate.* White solid; ^1^H-NMR (400 MHz, CDCl_3_) δ 8.48 (d, *J* = 4.6 Hz, 1H), 7.80 (d, *J* = 7.9 Hz, 1H), 7.67 (t, *J* = 7.8 Hz, 1H), 7.51–7.44 (m, 3H), 7.42–7.37 (m, 2H), 7.19–7.11 (m, 1H), 4.93 (s, 2H), 1.39 (s, 9H); ^13^C-NMR (100 MHz, CDCl_3_) δ 165.5, 150.5, 149.5, 144.2, 136.4, 136.3, 130.1, 129.7, 128.8, 127.4, 122.4, 121.7, 83.5, 50.1, 27.8; MS-ESI (*m*/*z*): calcd. for C_19_H_21_N_4_O_2_ [M + H]^+^ 337.2, found 337.2.

*tert-Butyl 2-(4,5-diphenyl-1H-1,2,3-triazol-1-yl)acetate.* White solid; ^1^H-NMR (400 MHz, CDCl_3_) δ 7.57 (d, *J* = 7.4 Hz, 2H), 7.54–7.44 (m, 3H), 7.35 (d, *J* = 7.1 Hz, 2H), 7.29–7.27 (m, 2H), 4.90 (s, 2H), 1.40 (s, 9H); ^13^C-NMR (100 MHz, CDCl_3_) δ 165.6, 144.4, 134.5, 130.9, 130.0, 130.0, 129.4, 128.5, 127.8, 127.6, 126.9, 83.5, 50.1, 27.9; MS-ESI (*m*/*z*): calcd. for C_20_H_22_N_3_O_2_ [M + H]^+^ 336.2, found 336.2.

*tert-Butyl 2-(4-(4-methoxyphenyl)-5-phenyl-1H-1,2,3-triazol-1-yl)acetate.* Orange solid; ^1^H-NMR (400 MHz, CDCl_3_) δ 7.55–7.44 (m, 5H), 7.34 (dd, *J* = 7.4, 2.1 Hz, 2H), 6.81 (d, *J* = 8.7 Hz, 2H), 4.89 (s, 2H), 3.78 (s, 3H), 1.39 (s, 9H); ^13^C-NMR (100 MHz, CDCl_3_) δ 165.8, 159.4, 144.4, 133.8, 130.1, 129.9, 129.4, 128.3, 127.8, 123.6, 114.0, 83.5, 55.3, 50.1, 28.0; MS-ESI (*m*/*z*): calcd for C_21_H_24_N_3_O_3_ [M + H]^+^ 366.2, found 366.2.

*tert-Butyl 2-(5-phenyl-4-(2-(trifluoromethyl)phenyl)-1H-1,2,3-triazol-1-yl)acetate.* Red solid; ^1^H-NMR (400 MHz, CDCl_3_) δ 7.72 (dd, *J* = 6.2, 3.0 Hz, 1H), 7.52–7.42 (m, 2H), 7.40–7.28 (m, 4H), 7.24–7.16 (m, 2H), 5.02 (s, 2H), 1.40 (s, 9H); ^13^C-NMR (100 MHz, CDCl_3_) δ 165.5, 143.1, 136.3, 133.3, 131.9, 131.5, 129.7, 129.5, 129.0, 128.9, 126.6 (q, *J* = 5.1 Hz), 126.5, 125.2, 122.5, 83.7, 50.6, 27.9; MS-ESI (*m*/*z*): calcd. for C_21_H_21_F_3_N_3_O_2_ [M + H]^+^ 404.2, found 404.2.

*tert-Butyl 2-(5-phenyl-4-(pyridin-4-yl)-1H-1,2,3-triazol-1-yl)propanoate.* Yellow solid; ^1^H-NMR (400 MHz, CDCl_3_) δ 8.52 (s, 2H), 7.82 (s, 2H), 7.72–7.61 (m, 2H), 7.58–7.47 (m, 1H), 7.36 (d, *J* = 6.8 Hz, 1H), 4.83 (q, *J* = 7.3 Hz, 1H), 1.91 (d, *J* = 7.3 Hz, 3H), 1.41 (s, 9H); ^13^C-NMR (100 MHz, CDCl_3_) δ 168.1, 150.1, 141.6, 138.6, 136.0, 130.6, 130.0, 129.8, 127.1, 120.7, 83.4, 57.1, 27.9, 17.0; MS-ESI (*m*/*z*): calcd. for C_20_H_23_N_4_O_2_ [M + H]^+^ 351.2, found 351.2.

*tert-Butyl 2-(5-phenyl-4-(pyridin-4-yl)-1H-1,2,3-triazol-1-yl)butanoate.* White solid; ^1^H-NMR (400 MHz, CDCl_3_) δ 8.52 (s, 2H), 7.89 (s, 2H), 7.77–7.61 (m, 3H), 7.40–7.31 (m, 2H), 4.58 (dd, *J* = 10.7, 4.7 Hz, 1H), 2.51 (ddq, *J* = 14.5, 10.6, 7.3 Hz, 1H), 2.34 (dqd, *J* = 14.7, 7.4, 4.6 Hz, 1H), 1.42 (s, 9H), 0.91 (t, *J* = 7.4 Hz, 3H); ^13^C-NMR (100 MHz, CDCl_3_) δ 167.6, 150.2, 141.4, 138.6, 136.7, 130.6, 130.1, 129.8, 127.2, 120.7, 83.3, 63.0, 28.0, 24.2, 10.9; MS-ESI (*m*/*z*): calcd. for C_21_H_25_N_4_O_2_ [M + H]^+^ 365.2, found 365.2.

## Figures and Tables

**Table 1:**
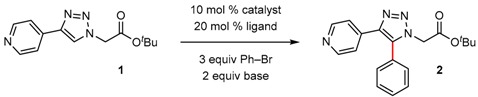
Optimization of palladium-catalyzed C–H arylation of **1**.

Entry	Catalyst	Ligand	Base	Temperature	Time	Solvent	Yield
1	CuI	–	*t*-BuLi	120 °C	24 h	DMF	10% ^a^
2	Pd(OAc)_2_	–	*n*-Bu_4_NOAc	120 °C	24 h	NMP	15% ^a^
3	Pd(OAc)_2_	–	*n*-Bu_4_NOAc	120 °C	20 h	DMF	21% ^a^
4	Pd(OAc)_2_	–	Cs_2_CO_3_	120 °C	20 h	DMF	6% ^a^
5	Pd(OAc)_2_	–	K_2_CO_3_	120 °C	20 h	DMF	31% ^a^
6	Pd(OAc)_2_	PPh_3_	K_2_CO_3_	120 °C	20 h	DMF	75% ^a^ 68% ^b^
7	Pd(OAc)_2_	P(*o*-Tol)_3_	K_2_CO_3_	120 °C	20 h	DMF	70% ^a^
8	Pd(OAc)_2_	PPh_3_	K_2_CO_3_	100 °C	24 h	DMF	77% ^a^
9	Pd(OAc)_2_	P(*n*-Bu)_3_	K_2_CO_3_	100 °C	24 h	DMF	<5% ^a^
10	Pd(OAc)_2_	PCy_3_	K_2_CO_3_	100 °C	24 h	DMF	20% ^a^
11	Pd(OAc)_2_	P(2-furyl)_3_	K_2_CO_3_	100 °C	24 h	DMF	29% ^a^
12	Pd(OAc)_2_	Cy-JohnPhos	K_2_CO_3_	100 °C	24 h	DMF	19% ^a^
13	Pd_2_(dba)_3_^c^		K_2_CO_3_	100 °C	24 h	DMF	7% ^a^
14	Pd(OAc)_2_	PPh_3_	K_2_CO_3_	120 °C	20 h	toluene	95% ^a^ 89% ^b^

^a^ Estimated by HPLC; ^b^ Isolated yield; ^c^ 5 mol % catalyst.

**Table 2:**
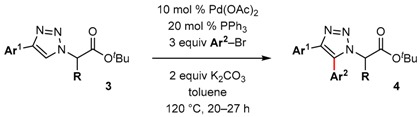
Scope of palladium-catalyzed C–H arylation of triazoles.

Entry	Ar^1^	Ar^2^	R	Yield
1	4-pyridyl	phenyl	H	89%
2	4-pyridyl	4-MeO-phenyl	H	85%
3	4-pyridyl	4-EtO_2_C-phenyl	H	92%
4	4-pyridyl	4-F_3_C-phenyl	H	83%
5	4-pyridyl	4-NC-phenyl	H	79%
6	4-pyridyl	4-F-phenyl	H	51%
7	4-pyridyl	3-Me-phenyl	H	86%
8	4-pyridyl	3-OHC-phenyl	H	32%
9	4-pyridyl	2-MeO-phenyl	H	82%
10	4-pyridyl	2-Me-phenyl	H	49%
11	4-pyridyl	1-naphthyl	H	78%
12	4-pyridyl	phenyl	H	80%
13	4-pyridyl	phenyl	H	84%
14	phenyl	phenyl	H	80%
15	4-MeO-phenyll	phenyl	H	64%
16	2-F_3_C-phenyl	phenyl	H	50%
17	4-pyridyl	phenyl	Me	20% ^a^
18	4-pyridyl	phenyl	Et	8% ^a^

^a^ Microwave heating, 140 °C, 15 min.

## Abbreviations

The following abbreviations are used in this manuscript:

Cy-JohnPhos: (2-Biphenyl)dicyclohexylphosphine
dba: dibenzylideneacetone
DMF: *N*,*N*-Dimethylformamide
DMSO: Dimethyl sulfoxide
NMP: *N*-Methylpyrrolidine

## References

[1] Kolb H.C., Sharpless K.B. (2003). The growing impact of click chemistry on drug discovery. Drug Discov. Today.

[2] Agalave S.G., Maujan S.R., Pore V.S. (2011). Click chemistry: 1,2,3-triazoles as pharmacophores. Chem. Asian J..

[3] Mosesa J.E., Moorhouse A.D. (2007). The growing applications of click chemistry. Chem. Soc. Rev..

[4] Lauria A., Mingoia R.D.F., Terenzi A., Martorana A., Barone G., Almerico A.M. (2014). 1,2,3-triazole in heterocyclic compounds, endowed with biological activity, through 1,3-dipolar cycloadditions. Eur. J. Org. Chem..

[5] Angella Y.L., Burgess K. (2007). Peptidomimetics via copper-catalyzed azide–alkyne cycloadditions. Chem. Soc. Rev..

[6] Hawker C.J., Wooley K.L. (2005). The convergence of synthetic organic and polymer chemistries. Science.

[7] Lutz J.-F. (2007). 1,3-dipolar cycloadditions of azides and alkynes: A universal ligation tool in polymer and materials science. Angew. Chem. Int. Ed..

[8] Rostovtsev V.V., Green L.G., Fokin V.V., Sharpless K.B. (2002). A stepwise huisgen cycloaddition process: Copper(I)-catalyzed regioselective “ligation” of azides and terminal alkynes. Angew. Chem. Int. Ed..

[9] Tornøe C.W., Christensen C., Meldal M. (2002). Peptidotriazoles on solid phase: [1,2,3]-triazoles by regiospecific copper(I)-catalyzed 1,3-dipolar cycloadditions of terminal alkynes to azides. J. Org. Chem..

[10] Zhang L., Chen X., Xue P., Sun H.H.Y., Williams I.D., Sharpless K.B., Fokin V.V., Jia G. (2005). Ruthenium-catalyzed cycloaddition of alkynes and organic azides. J. Am. Chem. Soc..

[11] Bock V.D., Hiemstra H., van Maarseveen J.H. (2006). CuI-catalyzed alkyne–azide “click” cycloadditions from a mechanistic and synthetic perspective. Eur. J. Org. Chem..

[12] Hein J.E., Fokin V.V. (2010). Copper-catalyzed azide–alkyne cycloaddition (CuAAC) and beyond: New reactivity of copper(I) acetylides. Chem. Soc. Rev..

[13] Totobenazara J., Burke A.J. (2015). New click-chemistry methods for 1,2,3-triazoles synthesis: Recent advances and applications. Tetrahedron Lett..

[14] Singh M.S., Chowdhury S., Koley S. (2016). Advances of azide-alkyne cycloaddition-click chemistry over the recent decade. Tetrahedron.

[15] Krasiński A., Fokin V.V., Sharpless K.B. (2004). Direct synthesis of 1,5-disubstituted-4-magnesio-1,2,3-triazoles, revisited. Org. Lett..

[16] Majireck M.M., Weinreb S.M. (2006). A study of the scope and regioselectivity of the ruthenium-catalyzed [3 + 2]-cycloaddition of azides with internal alkynes. J. Org. Chem..

[17] Boren B.C., Narayan S., Rasmussen L.K., Zhang L., Zhao H., Lin Z., Jia G., Fokin V.V. (2008). Ruthenium-catalyzed azide-alkyne cycloaddition: Scope and mechanism. J. Am. Chem. Soc..

[18] Hein J.E., Tripp J.C., Krasnova L.B., Sharpless K.B., Fokin V.V. (2009). Copper(I)-catalyzed cycloaddition of organic azides and 1-iodoalkynes. Angew. Chem. Int. Ed..

[19] Meza-Aviña M.E., Patel M.K., Lee C.B., Dietz T.J., Croatt M.P. (2011). Selective formation of 1,5-substituted sulfonyl triazoles using acetylides and sulfonyl azides. Org. Lett..

[20] Smith C.D., Greaney M.F. (2013). Zinc mediated azide-alkyne ligation to 1,5- and 1,4,5-substituted 1,2,3-triazoles. Org. Lett..

[21] Wei F., Li H., Song C., Ma Y., Zhou L., Tung C.-H., Xu Z. (2015). Cu/Pd-catalyzed, three-component click reaction of azide, alkyne, and aryl halide: One-pot strategy toward trisubstituted triazoles. Org. Lett..

[22] Chuprakov S., Chernyak N., Dudnik A.S., Gevorgyan V. (2007). Direct Pd-catalyzed arylation of 1,2,3-triazoles. Org. Lett..

[23] Ackermann L., Potukuchi H.K., Landsberg D., Vicente R. (2008). Copper-catalyzed “click” reaction/direct arylation sequence: Modular syntheses of 1,2,3-triazoles. Org. Lett..

[24] Ackermann L., Vicente R. (2009). Catalytic direct arylations in polyethylene glycol (PEG): Recyclable palladium(0) catalyst for C−H bond cleavages in the presence of air. Org. Lett..

[25] Ackermann L., Vicente R., Born R. (2008). Palladium-catalyzed direct arylations of 1,2,3-triazoles with aryl chlorides using conventional heating. Adv. Synth. Catal..

[26] Ackermann L., Althammer A., Fenner S. (2009). Palladium-catalyzed direct arylations of heteroarenes with tosylates and mesylates. Angew. Chem. Int. Ed..

[27] Liégault B., Lapointe D., Caron L., Vlassova A., Fagnou K. (2009). Establishment of broadly applicable reaction conditions for the palladium-catalyzed direct arylation of heteroatom-containing aromatic compounds. J. Org. Chem..

[28] Lu J., Tan X., Chen C. (2007). Palladium-catalyzed direct functionalization of imidazolinone: Synthesis of dibromophakellstatin. J. Am. Chem. Soc..

[29] Ryabov A.D. (1990). Mechanisms of intramolecular activation of C–H bonds in transition-metal complexes. Chem. Rev..

[30] Lapointe D., Fagnou K. (2010). Overview of the mechanistic work on the concerted metallation–deprotonation pathway. Chem. Lett..

[31] Gómez M., Granell J., Martinez M. (1997). Variable-temperature and -pressure kinetics and mechanism of the cyclopalladation reaction of imines in aprotic solvent. Organometallics.

[32] Biswas B., Sugimoto M., Sakaki S. (2000). C−H bond activation of benzene and methane by M(η2-O_2_CH)_2_ (M = Pd or Pt). A theoretical study. Organometallics.

[33] Kurzeev S.A., Kazankov G.M., Ryabov A.D. (2002). Second- and inverse order pathways in the mechanism of orthopalladation of primary amines. Inorg. Chim. Acta.

[34] Davies D.L., Donald S.M.A., Macgregor S.A. (2005). Computational study of the mechanism of cyclometalation by palladium acetate. J. Am. Chem. Soc..

[35] García-Cuadrado D., Braga A.A.C., Maseras F., Echavarren A.M. (2006). Proton abstraction mechanism for the palladium-catalyzed intramolecular arylation. J. Am. Chem. Soc..

[36] Lafrance M., Rowley C.N., Woo T.K., Fagnou K. (2006). Catalytic intermolecular direct arylation of perfluorobenzenes. J. Am. Chem. Soc..

[37] Lafrance M., Fagnou K. (2006). Palladium-catalyzed benzene arylation: Incorporation of catalytic pivalic acid as a proton shuttle and a key element in catalyst design. J. Am. Chem. Soc..

[38] Moradi W.A., Buchwald S.L. (2001). Palladium-catalyzed α-arylation of esters. J. Am. Chem. Soc..

[39] Jørgensen M., Lee S., Liu X., Wolkowski J.P., Hartwig J.F. (2002). Efficient synthesis of α-aryl esters by room-temperature palladium-catalyzed coupling of aryl halides with ester enolates. J. Am. Chem. Soc..

[40] Bellina F., Rossi R. (2010). Transition metal-catalyzed direct arylation of substrates with activated sp^3^-hybridized C–H bonds and some of their synthetic equivalents with aryl halides and pseudohalides. Chem. Rev..

